# Psychometric properties of the German version of the Eating Pathology Symptoms Inventory

**DOI:** 10.1186/s40337-025-01253-7

**Published:** 2025-04-07

**Authors:** Adrian Meule, Sebastian Ertl, Kelsie T. Forbush, Luka M. Mindrup, Johannes C. Ehrenthal, David R. Kolar

**Affiliations:** 1 Department of Psychology, University of Regensburg, Universitätsstraße 31, 93053 Regensburg, Germany; 2 Department of Psychology, University of Kansas, Lawrence, KS USA; 3 Department of Psychology, University of Cologne, Cologne, Germany

**Keywords:** Eating disorders, Anorexia nervosa, Bulimia nervosa, Binge eating disorder, Assessment

## Abstract

**Background:**

The Eating Pathology Symptoms Inventory (EPSI) is a multidimensional self-report measure for the assessment of eating pathology and related aspects: Body Dissatisfaction, Binge Eating, Cognitive Restraint, Purging, Restricting, Excessive Exercise, Negative Attitudes Toward Obesity, and Muscle Building. The aims of the current studies were to provide a German translation of the EPSI and replicate the original EPSI’s psychometric properties and correlates.

**Methods:**

In two cross-sectional surveys using convenience samples (*n* = 361 and *n* = 178), participants completed the German EPSI along with other questionnaires.

**Results:**

In both studies, the EPSI’s eight-factor structure, high internal consistencies, and differential associations with other instruments assessing eating disorder-specific and general psychopathology as well as gender differences on the EPSI’s scales were largely replicated.

**Conclusions:**

The German EPSI had sound psychometric properties that allow for a reliable and valid, multidimensional assessment of eating-disorder psychopathology.

## Abstract (plain language summary)

The Eating Pathology Symptoms Inventory (EPSI) is a questionnaire for measuring symptoms of eating disorders and related aspects. We translated the EPSI into German and demonstrate in two studies that its psychometric properties and correlates are comparable to the original English version and, thus, that it can be used by researchers and clinicians in German-speaking countries.

## Background

The Eating Pathology Symptoms Inventory (EPSI) is a multidimensional self-report measure for the assessment of eating-disorder psychopathology [1]. Development of the EPSI was motivated by the fact that other widely used self-report measures had several shortcomings. For example, the proposed factor structures of established instruments such as the Eating Disorder Examination–Questionnaire (EDE–Q), the Eating Attitudes Test (EAT), or the Eating Disorder Inventory (EDI) have received limited empirical support [2–6]. Moreover, past measures of eating-disorder psychopathology were developed in primarily female samples with anorexia nervosa and bulimia nervosa and, thus, did not fully cover aspects of eating disorders relevant for men such as muscle-building tendencies [7]. Furthermore, other measures (e.g., the EAT and EDI) focus on a restricted range of eating-disorder symptoms such as body dissatisfaction and restrained eating and do not cover other key symptoms such as excessive exercise.

The EPSI addresses these issues by assessing several distinct aspects of eating disorders via eight scales: *Body Dissatisfaction* (i.e., dissatisfaction with one’s weight and shape), *Binge Eating* (i.e., eating large amounts of food and subjective feelings of loss-of-control over eating), *Cognitive Restraint* (i.e., intentions to restrict food intake, regardless of amount consumed), *Purging* (i.e., behaviors designed to compensate for food consumption such as self-induced vomiting), *Restricting* (i.e., actual restriction of food intake), *Excessive Exercise* (i.e., exercising in a driven, compulsive, or excessive manner), *Negative Attitudes Toward Obesity* (i.e., negative viewpoints about persons with overweight or obesity), and *Muscle Building* (i.e., striving for high muscularity; [1]). This eight-factor structure has largely been replicated across different samples, for example, in both clinical and non-clinical samples, in both men and women, and in both adolescents and adults, with internal consistencies usually being greater than 0.80 across scales [8–11]. There are also several translated versions in languages such as Chinese [12], Farsi [13, 14], and Swedish [15] as well as a clinician-rated interview version [16].

Supporting convergent and discriminant validity of the EPSI, differential associations have been found between the EPSI’s scales and measures of eating disorder-specific and general psychopathology. For example, large correlations have been reported between EPSI scales assessing eating-disorder behaviors and cognitions and other measures of the same constructs, whereas scores on instruments assessing depression and anxiety symptoms were unrelated or weakly positively related to the EPSI across scales [1, 9]. Gender differences on the EPSI’s scales have been reported such that men tend to have higher scores than women on Excessive Exercise, Negative Attitudes Toward Obesity, and Muscle Building whereas women tend to have higher scores than men on all other scales, particularly the Body Dissatisfaction scale [9, 17].

Although there is a large range of eating-disorder questionnaires available in English, there are only few of such measures available in German. Specifically, one of the most often used eating-disorder questionnaires in German-speaking countries is the EDE–Q [18] but other available measures that comprehensively assess eating-disorder symptomatology are outdated (e.g., are not based on current diagnostic classification systems or have never been updated, for example, there is only a German version of the EDI–2 and not of the EDI–3, cf. ref [19]) or lack factorial validity and comprehensiveness, as described above. Moreover, translating existing measures that are already available in other languages potentially fosters cross-cultural research on eating disorders. Thus, the aim of the current studies was to provide a German translation of the EPSI and replicate the original EPSI’s psychometric properties and correlates in two convenience samples. Study 1 aimed to replicate the EPSI’s factor structure, internal consistencies, associations with other instruments assessing eating disorder-specific and general psychopathology, and gender differences, as described above. As “credibility of scientific claims is established with evidence for their replicability using new data” (ref [20], p. 1), Study 2 aimed to replicate these findings using different instruments assessing eating disorder-specific and general psychopathology according to preregistered analyses (https://osf.io/e8rjv).

## Study 1

### Methods

#### Participants

The study was approved by the ethics committee of the University of Regensburg, Regensburg, Germany (Reference no. 22-3111-101) as part of a larger international collaboration study on food insecurity in adult (≥ 18 years old) university students. Participants were recruited through an internal system for posting studies at the University of Regensburg, flyers and posters, and with social media posts. Participants who were psychology students at the University of Regensburg were offered course credits for their participation. Five-hundred and seventy-one persons visited the website but 170 did not start the survey, 14 did not meet inclusion criteria (one did not indicate their age and 13 indicated that they were not students), and 26 cancelled the survey before completing the EPSI. No participants were excluded due to conspicuous short completion times in the survey or otherwise suspicious entries. The 361 persons who completed the EPSI had a mean age of 21.5 years (*SD* = 3.29, Range: 18–47) and a mean body mass index of 21.8 kg/m² (*SD* = 3.26, Range: 15.9–40.6, data available for 340 persons). Most participants identified as female (*n* = 309 of 357, 86.6%, four participants selected *non-binary/don’t want to answer*) and had German citizenship (*n* = 336 of 355, 94.7%, six participants did not enter their nationality). The study was not preregistered.

#### Measures

*EPSI*. The EPSI [1] is a self-report questionnaire for measuring various aspects of eating-disorder psychopathology in the past four weeks. It has 45 items that are answered on a five-point scale ranging from 0 = *never* to 4 = *very often*. The EPSI has eight scales for measuring Body Dissatisfaction, Binge Eating, Cognitive Restraint, Purging, Restricting, Excessive Exercise, Negative Attitudes Toward Obesity, and Muscle Building. Item responses were averaged for each scale, which can, thus, range between 0 and 4 with higher scores representing higher eating pathology. An initial translation of the EPSI was conducted by a research student and discussed with DRK, followed by obtaining feedback from three separate focus groups (five female and five male university students in total). Additionally, feedback by two females with anorexia nervosa was incorporated. Finally, a back-translation was then commented by the original EPSI author and feedback was incorporated into the final German EPSI translation^1^.

*EDE–Q*. The EDE–Q [21] is a self-report questionnaire for measuring eating disorder psychopathology in the past four weeks and its German version [18] was used in the current study. It has 28 items, 22 of which are answered on a seven-point scale ranging from 0 = *no days*/*none of the times*/*not at all* to 6 = *every day*/*every time*/*markedly*. Four subscales (Eating Restraint, Shape Concern, Weight Concern, Eating Concern) have been proposed but these subscales have received little empirical support [3]. Thus, we only used the EDE–Q’s global score, for which all 22 items were averaged, which ranges from 0 to 6 with higher scores representing higher eating-disorder psychopathology. The remaining six items assessed the frequency of eating large amounts of food, loss-of-control eating, number of days on which binge eating episodes occurred, frequency of self-induced vomiting, use of laxatives, and excessive exercise.

*Body Shape Questionnaire–8 (BSQ–8)*. The BSQ–8 [22] is a self-report questionnaire for measuring concerns about body shape in the past four weeks. The German version of the BSQ–8 [23] was used in the current study. The BSQ–8 has eight items that are answered on a six-point scale ranging from 1 = *never* to 6 = *always*. Although sum scores were computed in the original validation studies, we used mean scores to be consistent across the different questionnaires used in the current study. Thus, total scores can range between 1 and 6 with higher scores representing higher body shape concerns.

*Depression Anxiety Stress Scales–21 (DASS–21)*. The DASS–21 [24] is a self-report questionnaire for measuring depression, anxiety, and stress in the past week. The German version of the DASS–21 [25] was used in the current study. The DASS–21 has 21 items that are answered on a four-point scale ranging from 0 = *did not apply to me at all* to 3 = *applied to me very much or most of the time*. Although sum scores were computed in the original validation studies, we used mean scores for each of the DASS–21’s three scales (Depression, Anxiety, Stress) to be consistent across the different questionnaires used in the current study. Thus, scale scores can range between 0 and 3 with higher scores representing higher depressive symptomatology, anxiety, and stress, respectively.

### Data analyses

Data were analyzed with R version 4.3.3 in RStudio version 2024.04.1. A confirmatory factor analysis was run with the *lavaan* package version 0.6–18, fitting the EPSI’s eight-factor structure. Similar to the original validation studies [1], weighted least squares mean and variance adjusted (WLSMV) was used as estimation method as it is a robust estimator for ordinal data [26, 27]. Model fit was evaluated with the Root Mean Square Error of Approximation (RMSEA), Comparative Fit Index (CFI), and Tucker–Lewis Index (TLI; also called non-normed fit index) according to the guidelines by Schermelleh-Engel and colleagues [28], who recommend interpreting RMSEA values between 0.05 and 0.08 and CFI/TLI values between 0.95 and 0.97 as indicating acceptable fit, and RMSEA values ≤ 0.05 and CFI/TLI values ≥ 0.97 as indicating good fit. Internal consistencies of the EPSI’s scales were examined with McDonald’s ω [29], obtained with the *psych* package version 2.4.6.26, as has been recommended [30].

Associations between the EPSI’s scales and the EDE–Q global score, number of binge days, number of times exercising excessively, BSQ–8 total score, and DASS–21 scale scores were examined with robust percentage bend correlation coefficients [31], obtained with the *WRS2* package version 1.1-6, as has been recommended [32]. Regarding the number of times of self-induced vomiting and use of laxatives, however, only 22 and 8 persons, respectively, indicated to have vomited or used laxatives at least once in the past 28 days and the *WRS2* package failed to compute the percentage bend correlation coefficients for these variables. As a workaround, we dichotomized these variables into 0 = *no vomiting*/*no use of laxatives* and 1 = *self-induced vomiting*/*use of laxatives at least once in the past 28 days* and computed rank-biserial correlation coefficients [33] with the *rstatix* package version 0.7.2.

Gender differences on the EPSI’s scales were tested with non-parametric Wilcoxon rank-sum tests (also called Mann–Whitney U test; [34]) with the *stats* package version 4.3.3. Rank-biserial correlation coefficients [33] were computed as effect sizes with the *rstatix* package version 0.7.2. Because of the large sample size and numerous inferential tests, we considered effects as significant when *p* <.005, as has been recommended by Benjamin and colleagues who argue that this alpha level “represents ‘substantial’ to ‘strong’ evidence according to conventional Bayes factor classifications” and “would reduce the false positive rate to levels we judge to be reasonable” (ref [35], p. 7). The data and code with which all results can be reproduced are available at https://osf.io/95fb3.

## Results

**Fig. 1:**
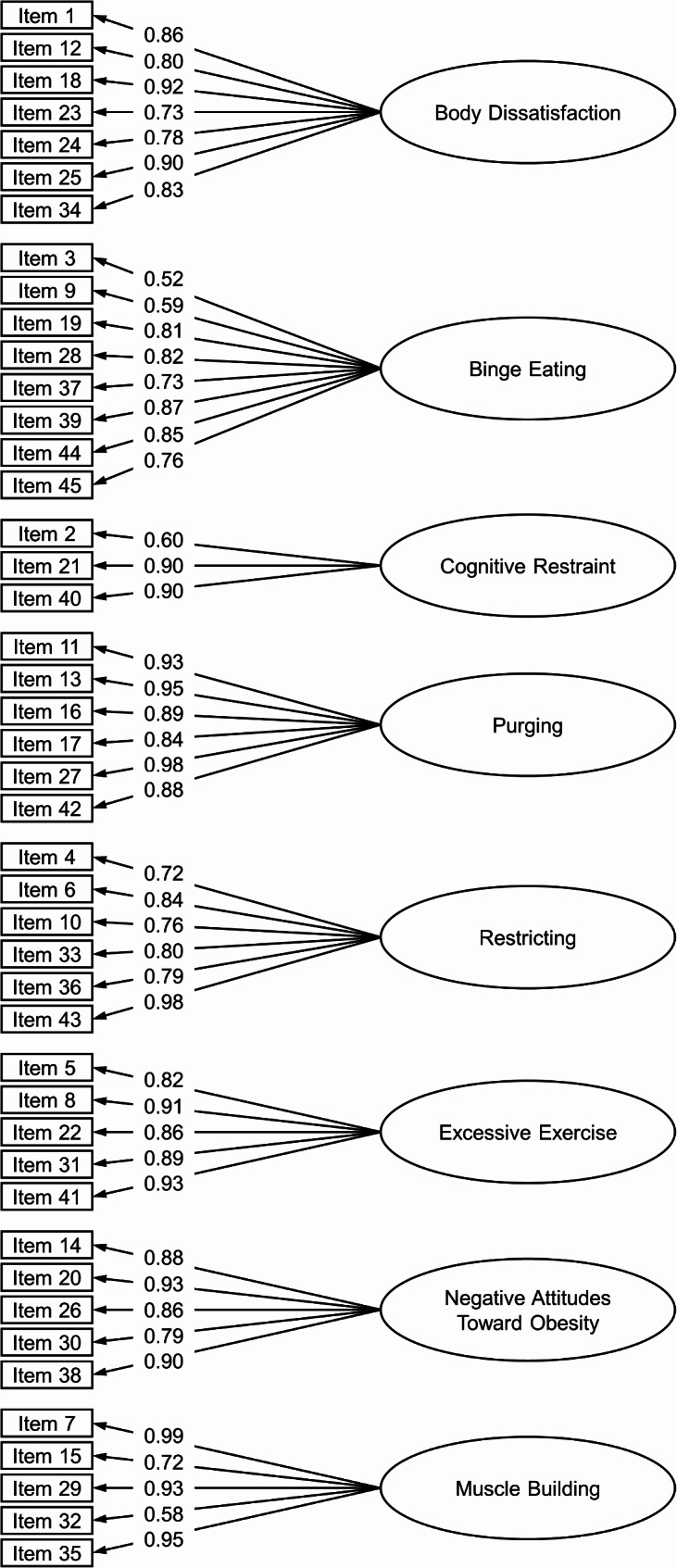
Standardized factor loadings for the EPSI scales in Study 1. For readability, variances and covariances are not displayed in this depiction. Covariances between scales can be found in Table 1

The eight-factor model demonstrated an acceptable-to-good fit to the data (RMSEA = 0.05, CFI = 0.96, TLI = 0.95). All standardized factor loadings were ≥ 0.52 (Fig. 1). All EPSI scales were significantly and positively correlated with each other, with few exceptions for the Negative Attitudes Toward Obesity and Muscle Building scale (Table 1). Internal consistencies were ω ≥ 0.83 for all scales (Table 1). Correlations between the EPSI and other measures are displayed in Table 2. The largest correlation with the EDE–Q global score was found with the Body Dissatisfaction scale. The largest correlation for the number of binge days was found with the Binge Eating scale. The largest correlation for self-induced vomiting as well as use of laxatives was found with the Purging scale. The largest correlation for the number of times exercising excessively was found with the Excessive Exercise scale. The largest correlation for the BSQ–8 was found with the Body Dissatisfaction scale. The largest correlations for the Depression, Anxiety, and Stress scales of the DASS–21 were found with the Body Dissatisfaction and Restricting scales. Women had higher Body Dissatisfaction scores than men, whereas men scored higher on Excessive Exercise, Negative Attitudes Toward Obesity, and Muscle Building. Women and men did not differ significantly on Binge Eating, Cognitive Restraint, Purging, and Restricting (Table 3).

**Table 1:** Descriptive statistics (*M*, *SD*), internal consistencies (ω), and standardized covariances of EPSI scales in study 1

*N* = 361	M (SD)	Body Dissatisfaction	Binge Eating	Cognitive Restraint	Purging	Restricting	Excessive Exercise	Negative Attitudes Toward Obesity	Muscle Building
Body Dissatisfaction	1.74 (0.98)	ω = 0.94	**0.63**	**0.69**	**0.77**	**0.50**	**0.28**	**0.16**	0.12
Binge Eating	1.27 (0.82)	**0.63**	ω = 0.91	**0.47**	**0.48**	**0.17**	**0.26**	**0.19**	**0.22**
Cognitive Restraint	1.71 (1.06)	**0.69**	**0.47**	ω = 0.83	**0.79**	**0.40**	**0.66**	**0.33**	**0.41**
Purging	0.20 (0.53)	**0.77**	**0.48**	**0.79**	ω = 0.93	**0.65**	**0.51**	**0.30**	0.13
Restricting	1.02 (0.90)	**0.50**	**0.17**	**0.40**	**0.65**	ω = 0.93	**0.18**	0.14	0.02
Excessive Exercise	1.17 (1.08)	**0.28**	**0.26**	**0.66**	**0.51**	**0.18**	ω = 0.93	**0.44**	**0.76**
Negative Attitudes Toward Obesity	0.96 (0.85)	**0.16**	**0.19**	**0.33**	**0.30**	0.14	**0.44**	ω = 0.93	**0.39**
Muscle Building	0.98 (0.96)	0.12	**0.22**	**0.41**	0.13	0.02	**0.76**	**0.39**	ω = 0.89

*Notes*. All standardized covariances > 0.14 are statistically significant at *p* <.005 (printed in bold)

**Table 2:** Descriptive statistics (*M*, *SD*) and internal consistencies (ω) of other measures and their correlations (*r*_pb_) with EPSI scales in study 1

	*n*	M (SD)	ω	Body Dissatisfaction	Binge Eating	Cognitive Restraint	Purging	Restricting	Excessive Exercise	Negative Attitudes Toward Obesity	Muscle Building
Eating Disorder Examination–Questionnaire											
Global score	354	1.67 (1.40)	0.97	**0.80**	**0.51**	**0.65**	**0.55**	**0.36**	**0.35**	**0.17**	**0.17**
Binge eating (number of days)	348	1.93 (3.99)	—	**0.44**	**0.64**	**0.31**	**0.31**	0.10	**0.16**	0.10	**0.17**
Self-induced vomiting (number of times)	347	0.42 (2.10)	—	**0.31**	**0.21**	**0.34**	**0.49**	**0.32**	**0.22**	0.05	0.02
Use of laxatives (number of times)	346	0.11 (0.91)	—	**0.21**	0.09	**0.19**	**0.34**	**0.19**	0.14	0.02	0.02
Excessive exercise (number of times)	347	1.73 (4.17)	—	**0.31**	**0.24**	**0.43**	**0.30**	**0.17**	**0.55**	0.14	**0.35**
Body Shape Questionnaire–8	344	2.51 (1.25)	0.96	**0.82**	**0.56**	**0.59**	**0.56**	**0.32**	**0.29**	**0.17**	0.13
Depression, Anxiety and Stress Scale–21											
Depression	356	0.89 (0.74)	0.95	**0.46**	**0.30**	**0.24**	**0.33**	**0.47**	0.11	0.13	0.07
Anxiety	356	0.72 (0.62)	0.86	**0.41**	**0.32**	**0.24**	**0.26**	**0.35**	0.07	0.13	0.09
Stress	356	1.03 (0.66)	0.90	**0.46**	**0.37**	**0.26**	**0.27**	**0.39**	0.09	0.10	0.03

*Notes*. All correlation coefficients > 0.15 are statistically significant at *p* <.005 (printed in bold). Note that percentage bend correlation coefficients could not be computed for self-induced vomiting and use of laxatives due to the small number of persons reporting these behaviors. Therefore, these variables were dichotomized into *no vomiting*/*no use of laxatives* versus *self-induced vomiting*/*use of laxatives at least once in the past 28 days* and rank-biserial correlation coefficients were computed. Note that—as other correlation coefficients—percentage bend and rank-biserial correlation coefficients can range between −1 and 1. Thus, their sizes may be interpreted as small (0.1–0.3), medium (0.3–0.5), or large (≥ 0.5), similar to what has been suggested for Pearson’s *r* [55]

**Table 3:** Descriptive and test statistics for gender differences on EPSI scales in study 1

*N* = 357	Male (*n* = 48) M (SD)	Female (*n* = 309) M (SD)	W	*p*	*r*_rb_
Body Dissatisfaction	1.21 (0.87)	1.81 (0.97)	4618	< 0.001	0.22
Binge Eating	1.23 (0.63)	1.27 (0.85)	7608	0.773	0.02
Cognitive Restraint	1.62 (0.93)	1.72 (1.08)	7164	0.703	0.02
Purging	0.08 (0.33)	0.22 (0.55)	6385	0.034	0.11
Restricting	0.77 (0.81)	1.05 (0.91)	5955	0.028	0.12
Excessive Exercise	1.69 (1.23)	1.08 (1.04)	9496	0.002	0.17
Negative Attitudes Toward Obesity	1.58 (0.91)	0.87 (0.80)	10,918	< 0.001	0.28
Muscle Building	1.62 (1.12)	0.88 (0.89)	10,401	< 0.001	0.24

*Notes*. Note that—as other correlation coefficients—rank-biserial correlation coefficients can range between −1 and 1. Thus, their sizes may be interpreted as small (0.1–0.3), medium (0.3–0.5), or large (≥ 0.5), similar to what has been suggested for Pearson’s *r* [55]

## Study 2

### Methods

#### Participants

The study was approved by the ethics committee of the Faculty of Human Sciences at the University of Cologne, Cologne, Germany (Reference no. JEHF0199). A convenience sample of adults was recruited through mailing lists at the University of Cologne (Cologne, Germany) and social media (https://www.instagram.com). Participants who were psychology students at the University of Cologne were offered course credits for their participation. One-hundred and fifty-six persons started the survey, and 149 persons completed the survey. We noticed that one participant had a suspicious response pattern (always selecting the same response category for each questionnaire) and a completion time of 74 s. Therefore, we removed the data for this participant before running further analyses (in contrast to the preregistration protocol, in which we stated that no data would be excluded; https://osf.io/e8rjv). The remaining 148 persons had a mean age of 27.4 years (*SD* = 12.1, Range: 18–84) and a mean body mass index of 22.8 kg/m² (*SD* = 4.18, Range: 16.5–40.4). Most participants identified as female (*n* = 104, 70.3%, no participant selected *non-binary*), had German citizenship (*n* = 139 of 147, 94.6%, data missing for one participant), and had higher school education (*n* = 96, 64.9%) or a university/college degree (*n* = 33, 22.3%). A minority of participants reported that they had ever received an eating-disorder diagnosis (*n* = 13, 8.78%) or had ever received eating-disorder treatment (*n* = 11, 7.43%).

#### Measures

*EPSI*. The German version of the EPSI as described in Study 1 was used.

*EDE–Q8*. The EDE–Q8 [36] is a short version of the EDE–Q for measuring eating disorder psychopathology in the past four weeks and its German version [18] was used in the current study. It has eight items that are answered on a seven-point scale ranging from 0 = *no days*/*none of the times*/*not at all* to 6 = *every day*/*every time*/*markedly*. All items were averaged to a total score, which can range between 0 and 6 with higher scores representing higher eating-disorder psychopathology.

*Commitment to Exercise Scale (CES)*. The CES [37] is a self-report questionnaire for measuring tendencies to exercise compulsively in general and its German version [38] was used in the current study. It has eight items that were answered in the original validation studies on a visual analogue scale anchored *not at all important*/*never upset*/*never*/*no routine*/*not at all* and *very important*/*always upset*/*always*/*strict routine*/*a great deal*. In the current study, however, we used a version with the same anchors but a four-point scale response format coded with 1–4, as has been used in other studies [39–43]. Moreover, this response format does not negatively affect the scales’ psychometric properties as a one-factor structure, high internal consistency, and high convergent validity with other measures of compulsive exercise could be demonstrated in a recent validation study in persons with eating disorders [44]. All items were averaged to a total score, which can range between 1 and 4 with higher scores representing stronger tendencies to exercise compulsively.

*Patient Health Questionnaire–4*. The PHQ–4 [45] is a self-report questionnaire for measuring depression and anxiety symptoms in the past two weeks and its German version [46] was used in the current study. It has four items that are answered on a four-point scale ranging from 0 = *not at all* to 3 = *nearly every day*. The PHQ–4 has two subscales (Depression and Anxiety), but we only analyzed the total score for parsimony.^2^ Although sum scores were computed in the original validation studies, we used mean scores to be consistent across the different questionnaires used in the current study. Thus, total scores can range between 0 and 3 with higher scores representing higher depression and anxiety symptomatology.

*Intolerance of Uncertainty Scale (IUS)*. The IUS [47] is a self-report questionnaire for measuring emotional, cognitive, and behavioral reactions to ambiguous situations, implications of being uncertain, and attempts to control the future. The German version [48] was used in the current study, which has 18 items that are answered on a five-point scale ranging from 1 = *not at all characteristic of me* to 5 = *entirely characteristic of me*. We averaged all items to a total score, which can range between 1 and 5 with higher scores representing higher intolerance of uncertainty.

### Data analyses

Data analyses were preregistered at https://osf.io/e8rjv and similar to Study 1 except that we used the EDE–Q8, CES, PHQ–4, and IUS instead of the EDE–Q, BSQ–8, and DASS–21 to examine correlates of the EPSI. The data and code with which all results can be reproduced are available at https://osf.io/95fb3.

## Results

**Fig. 2:**
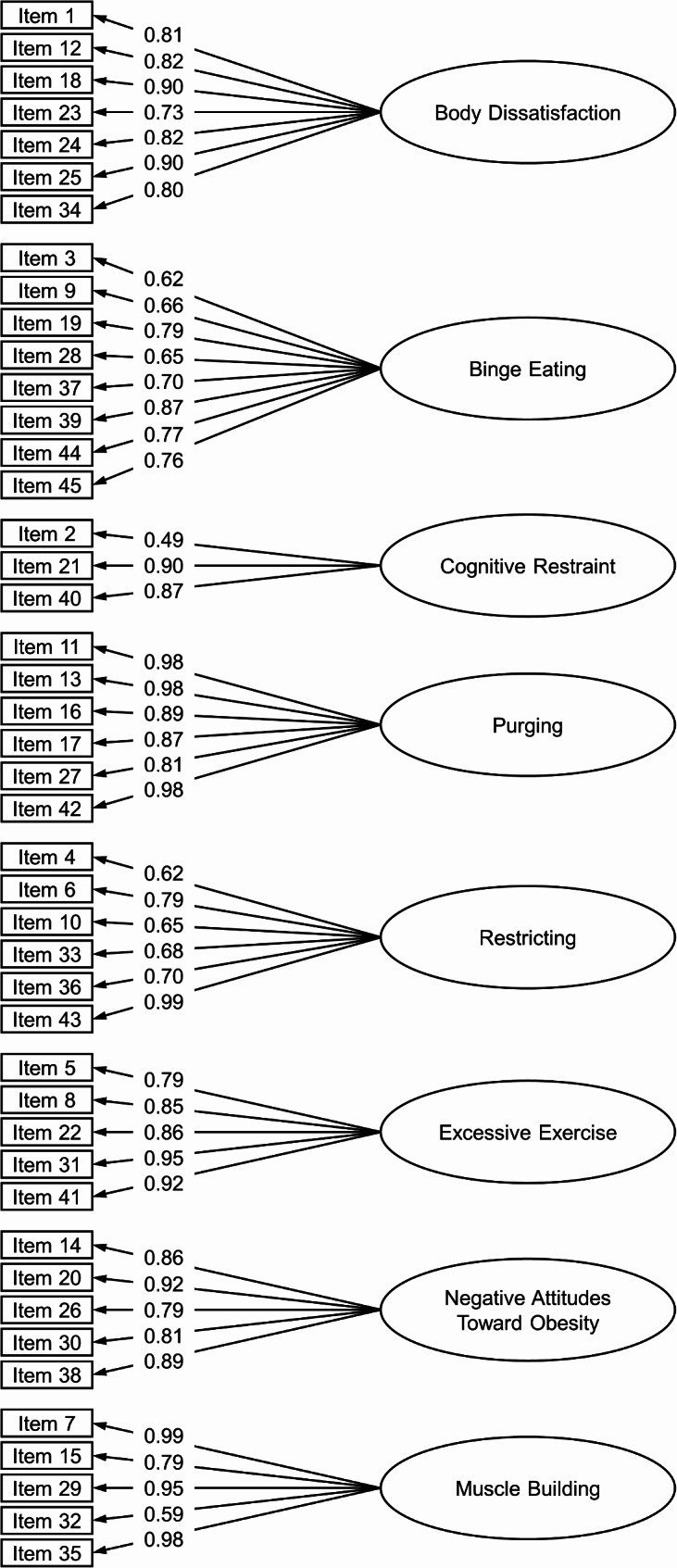
Standardized factor loadings for the EPSI scales in Study 2. For readability, variances and covariances are not displayed in this depiction. Covariances between scales can be found in Table 4

The eight-factor model had an acceptable fit to the data (RMSEA = 0.06, CFI = 0.96, TLI = 0.96). All standardized factor loadings were ≥ 0.49 (Fig. 2). All EPSI scales were significantly and positively correlated with each other, with few exceptions for the Restricting, Negative Attitudes Toward Obesity, and Muscle Building scale (Table 4). Internal consistencies were ω ≥ 0.77 for all scales (Table 4). Correlations between the EPSI and other measures are displayed in Table 5. The largest correlation for the EDE–Q8 was found with the Body Dissatisfaction scale. The largest for the CES was found with the Excessive Exercise scale. The largest correlations for the PHQ–4 and IUS were found with the Body Dissatisfaction scale. Women had higher Body Dissatisfaction and Purging scores than men, whereas men scored higher on Negative Attitudes Toward Obesity and Muscle Building. Women and men did not differ significantly on Binge Eating, Cognitive Restraint, Restricting, and Excessive Exercise (Table 6).

**Table 4:** Descriptive statistics (*M*, *SD*), internal consistencies (ω), and standardized covariances of EPSI scales in study 2

*N* = 148	M (SD)	Body Dissatisfaction	Binge Eating	Cognitive Restraint	Purging	Restricting	Excessive Exercise	Negative Attitudes Toward Obesity	Muscle Building
Body Dissatisfaction	1.74 (0.98)	ω = 0.94	**0.54**	**0.59**	**0.70**	**0.44**	**0.27**	0.15	0.06
Binge Eating	1.34 (0.80)	**0.54**	ω = 0.90	**0.43**	**0.66**	0.15	**0.32**	**0.25**	0.17
Cognitive Restraint	1.70 (1.04)	**0.59**	**0.43**	ω = 0.77	**0.76**	**0.39**	**0.63**	**0.32**	**0.41**
Purging	0.22 (0.55)	**0.70**	**0.66**	**0.76**	ω = 0.96	**0.50**	**0.23**	0.17	**0.18**
Restricting	0.92 (0.77)	**0.44**	0.15	**0.39**	**0.50**	ω = 0.91	0.09	0.16	0.05
Excessive Exercise	1.32 (1.12)	**0.27**	**0.32**	**0.63**	**0.23**	0.09	ω = 0.93	**0.46**	**0.75**
Negative Attitudes Toward Obesity	1.38 (0.93)	0.15	**0.25**	**0.32**	0.17	0.16	**0.46**	ω = 0.93	**0.49**
Muscle Building	1.06 (1.03)	0.06	0.17	**0.41**	**0.18**	0.05	**0.75**	**0.49**	ω = 0.89

*Notes*. All standardized covariances > 0.17 are statistically significant at *p* <.005 (printed in bold)

**Table 5:** Descriptive statistics (*M*, *SD*) and internal consistencies (ω) of other measures and their correlations (*r*_pb_) with EPSI scales in study 2

*N* = 148	M (SD)	ω	Body Dissatisfaction	Binge Eating	Cognitive Restraint	Purging	Restricting	Excessive Exercise	Negative Attitudes Toward Obesity	Muscle Building
Eating Disorder Examination–Questionnaire–8	1.89 (1.55)	0.96	**0.79**	**0.43**	**0.61**	**0.58**	**0.26**	**0.24**	0.14	0.09
Commitment to Exercise Scale	2.08 (0.66)	0.90	**0.25**	0.18	**0.34**	0.02	0.01	**0.64**	**0.29**	**0.42**
Patient Health Questionnaire–4	0.95 (0.68)	0.89	**0.49**	0.18	0.13	**0.24**	**0.23**	−0.04	0.08	−0.06
Intolerance of Uncertainty Scale	2.59 (0.85)	0.95	**0.49**	0.14	**0.29**	**0.37**	**0.32**	0.02	0.04	−0.03

*Notes*. All correlation coefficients > 0.22 are statistically significant at *p* <.005 (printed in bold). Note that—as other correlation coefficients—percentage bend correlation coefficients can range between −1 and 1. Thus, their sizes may be interpreted as small (0.1–0.3), medium (0.3–0.5), or large (≥ 0.5), similar to what has been suggested for Pearson’s *r* [55]

**Table 6:** Descriptive and test statistics for gender differences on EPSI scales in study 2

*N* = 148	Male (*n* = 44) M (SD)	Female (*n* = 104) M (SD)	W	*p*	*r*_rb_
Body Dissatisfaction	1.20 (0.80)	1.97 (0.97)	1195	< 0.001	0.38
Binge Eating	1.22 (0.73)	1.40 (0.83)	1996	0.220	0.10
Cognitive Restraint	1.62 (0.89)	1.74 (1.09)	2139	0.532	0.05
Purging	0.06 (0.43)	0.28 (0.59)	1565	< 0.001	0.33
Restricting	0.75 (0.57)	1.00 (0.83)	1959	0.166	0.11
Excessive Exercise	1.66 (1.14)	1.17 (1.08)	2888	0.012	0.21
Negative Attitudes Toward Obesity	1.97 (0.99)	1.12 (0.78)	3409	< 0.001	0.39
Muscle Building	1.61 (1.11)	0.83 (0.91)	3239	< 0.001	0.33

*Notes*. Note that—as other correlation coefficients—rank-biserial correlation coefficients can range between −1 and 1. Thus, their sizes may be interpreted as small (0.1–0.3), medium (0.3–0.5), or large (≥ 0.5), similar to what has been suggested for Pearson’s *r* [55]

## Discussion

The current study aimed to provide a German translation of the EPSI and replicate the original EPSI’s psychometric properties and correlates. Indeed, the EPSI’s eight-factor structure showed an acceptable fit to the data in both studies and internal consistencies across scales mostly were larger than 0.80, in line with previous studies [1, 8–11]. Results also supported convergent and discriminant validity of the German EPSI. For example, the EDE–Q and EDE–Q8 showed the largest correlations with the Body Dissatisfaction and Cognitive Restraint scale, in line with other studies that also used the EDE–Q and also found the largest correlations with these scales both in samples that were similar to the current samples (e.g., students) and in persons with eating disorders [1, 9]. Moreover, a similar pattern of results has been reported with the Swedish translation of the EPSI in adolescents [15]. This suggests that the current findings may likely translate to other samples such as more diverse community samples, persons with eating disorders, and samples across different countries. While there were more medium-sized associations with the Binge Eating and Purging scale, associations with the global EDE–Q score were mostly small for the other EPSI scales, suggesting that the global EDE–Q score primarily measures body dissatisfaction and related aspects and does not fully cover a wide range of eating disorder symptoms.

Examining specific eating disorder-related cognitions and behaviors revealed the largest correlations between body shape concerns and the Body Dissatisfaction scale, between binge eating frequency and the Binge Eating scale, between purging frequency (self-induced vomiting and use of laxatives) and the Purging scale, and between excessive exercising frequency (Study 1)/compulsive exercise (Study 2) and the Excessive Exercise scale, supporting both convergent and discriminant validity of these scales. Instruments assessing general psychopathology (depression, anxiety, stress) showed low-to-moderate correlations across the EPSI’s scales, also replicating prior findings [1, 9]. Low-to-moderate correlations across scales were also found for intolerance of uncertainty in Study 2, which somewhat dovetails with a study that found that most EPSI scales—except the Muscle Building scale—were weakly, positively correlated with perfectionism [49]. The largest correlation for intolerance of uncertainty was found with the EPSI’s Body Dissatisfaction subscale, which is in line with studies that examined intolerance of uncertainty in persons with eating disorders [50, 51].

Gender differences on the EPSI’s scales were inconsistent across Study 1 and Study 2, which may be due to different sample sizes and relatively few male participants in both studies. However, the largest effect sizes for gender-based group differences were found for Body Dissatisfaction, Negative Attitudes Toward Obesity, and Muscle Building scales. Specifically, females reported higher body dissatisfaction than males and males reported more negative attitudes towards obesity and higher muscle building tendencies than females. This pattern of results is largely in line with other studies that investigated both similar (e.g., students) and different (e.g., military recruits) samples [9, 17] as well as with a study that used the Chinese translation of the EPSI [12]. Yet, somewhat different findings have been reported with the Farsi translation of the EPSI in Iranian adolescents and adults (e.g., no gender differences in negative attitudes towards obesity, cf. refs [13, 14])., suggesting that there might be indeed be some cultural differences regarding gender differences on the EPSI’s scales.

As in every study, interpretation of results is limited to the samples studied—in this case, mostly young, highly educated, German adults without eating disorders—and, thus, may not translate to samples with different characteristics. Our sample composition also did not allow for tests of measurement invariance across different groups (e.g., gender, age groups), as has been done in other studies [1, 9, 11]. Furthermore, while findings generally supported convergent validity of EPSI scales, we did not use a complementary measure of muscle dysmorphia to explicitly test convergent validity of the Muscle Building scale. Other limitations pertain to the use of self-report measures and the cross-sectional design of the studies. Specifically, self-report measures can potentially be biased (e.g., by demand effects, social desirability, or recall bias) and cross-sectional designs do not allow for inferring causal relationships between variables. Thus, future avenues for further examining validity and clinical utility of the German EPSI would be to use it in more diverse samples, clinical samples, and in longitudinal or treatment studies. Such studies would also allow to examine further psychometric properties such as test–retest reliability or to derive cut-off scores that discriminate between persons with and without eating disorders. While we do provide German reference values using data from the combined samples on the EPSI’s website (https://care.ku.edu/epsi), which might be helpful for researchers and practitioners who want to use the German version, these numbers should be considered tentative as they do not stem from a sample representative for the German population or representative for persons with eating disorders.

Other future directions that pertain to the EPSI more generally might be to examine its potential to overlook certain eating-disorder symptoms. For example, while the EPSI covers a wide range of aspects, it still focuses on symptoms relevant to anorexia nervosa, bulimia nervosa, and binge-eating disorder. That is, it does not include a scale for assessing pica and rumination disorder. Moreover, persons with avoidant/restrictive food intake disorder (ARFID) might achieve high scores on the Restricting scale [52]. Yet, as this scale does not ask for different motivations underlying this restriction (e.g., lack of interest in eating or food, avoidance based on the sensory characteristics of food, concern about aversive consequences of eating), it may not differentiate well between persons with ARFID and persons with other eating disorders such as anorexia nervosa. On the other hand, by considering all of the EPSI’s scales, differentiating between persons with ARFID and persons with other eating disorders may indeed be possible (e.g., as persons with ARFID might score high on the Restricting but low on other scales, in contrast to persons with other eating disorders, cf. ref [52]). Another future direction may the examination of cross-cultural differences. While the EPSI is now available in four languages other than English, it is still not available in other widely-spoken languages such as Spanish, Hindi, Portuguese, Bengali, Russian, Japanese, Arabic, or French. Thus, future studies may provide such translations and examine the relevance of the EPSI across different languages and cultural contexts (e.g., non-Western populations).

In conclusion, the current studies largely replicated the EPSI’s psychometric properties and correlates in terms of factor structure, internal reliability, convergent and discriminant validity as well as gender differences. Thus, the German version of the EPSI had sound psychometric properties in two non-clinical samples, suggesting that it may be used as a reliable and valid, multidimensional assessment of eating pathology symptoms and related aspects.

## Data Availability

The data and code with which all results can be reproduced can be accessed at https://osf.io/95fb3.

## Footnotes

1 From “Development and validation of the Eating Pathology Symptoms Inventory,” by Forbush, K. T., et al. 2013, Psychological Assessment, 25, 859-878. Copyright © 2011 by Kelsie T. Forbush. Reproduced with permission. No further reproduction, modification, or distribution of the Eating Pathology Symptoms Inventory, derivative versions, or translated versions is permitted without advance, written permission from the copyright holder (Dr. Kelsie Forbush).

2 One of the reviewers asked for a further justification why only the total score of the PHQ–4 was analyzed. One reason is that findings in Study 1 indicated that correlations with the EPSI’s scales are basically similar for depression and anxiety. Moreover, we further conducted a Haberman analysis [53] for the PHQ–4 with the R package *subscore* [54]. This package computes the proportional reduction in mean squared error (PRMSE), which was larger for the total score than for the depression subscale (PRMSE.x = 0.81 vs. PRMSE.s = 0.75) and the anxiety subscale (PRMSE.x = 0.83 vs. PRMSE.s = 0.82), thus indicating that reporting the subscale scores does not have added value to reporting the total score [54]. The analytical code for this can be accessed at https://osf.io/95fb3.

## References

[1] Forbush KT, Wildes JE, Pollack LO, Dunbar D, Luo J, Patterson K, et al. Development and validation of the eating pathology symptoms inventory (EPSI). Psychol Assess. 2013;25(3):859–78. 23815116 10.1037/a0032639

[2] Barstack S, Karkhanis S, Erford BT, Bennett E, Buchanan E, Sharpe C, et al. Synthesis of the eating disorder Inventory-Third edition (EDI-3) psychometric characteristics: implications for counseling practice and research. J Couns Dev. 2023;101(3):359–72.

[3] Rand-Giovannetti D, Cicero DC, Mond JM, Latner JD. Psychometric properties of the eating disorder Examination–Questionnaire (EDE-Q): a confirmatory factor analysis and assessment of measurement invariance by sex. Assessment. 2020;27:164–77. 29094603 10.1177/1073191117738046

[4] Papini NM, Jung M, Cook A, Lopez NV, Ptomey LT, Herrmann SD, et al. Psychometric properties of the 26-item eating attitudes test (EAT-26): an application of Rasch analysis. J Eat Disorders. 2022;10:62. 10.1186/s40337-022-00580-3 PMC9069796 35509106

[5] Ocker LB, Lam ETC, Jensen BE, Zhang JJ. Psychometric properties of the eating attitudes test. Meas Phys Educ Exerc Sci. 2007;11(1):25–48.

[6] Jenkins PE, Rienecke RD. Structural validity of the eating disorder Examination—Questionnaire: a systematic review. Int J Eat Disord. 2022;55(8):1012–30. 35503783 10.1002/eat.23721 PMC9543786

[7] Christensen Pacella KA, Wossen L, Hagan KE. Low overlap and high heterogeneity across common measures of eating disorder pathology: a content analysis. Assessment. 2024;32:48–60. 10.1177/10731911241238084 38519835

[8] Coniglio KA, Becker KR, Tabri N, Keshishian AC, Miller JD, Eddy KT, et al. Factorial integrity and validation of the eating pathology symptoms inventory (EPSI). Eat Behav. 2018;31:1–7. 30025234 10.1016/j.eatbeh.2018.07.004

[9] Forbush KT, Wildes JE, Hunt TK. Gender norms, psychometric properties, and validity for the eating pathology symptoms inventory. Int J Eat Disord. 2014;47(1):85–91. 23996154 10.1002/eat.22180

[10] Perko VL, Forbush KT, Christensen KA, Richson BN, Chapa DAN, Bohrer BK, et al. Validation of the factor structure of the eating pathology symptoms inventory in an international sample of sexual minority men. Eat Behav. 2021;42:101511. 34004456 10.1016/j.eatbeh.2021.101511 PMC10042082

[11] Richson BN, Forbush KT, Chapa DAN, Gould SR, Perko VL, Johnson SN, et al. Measurement invariance of the eating pathology symptoms inventory (EPSI) in adolescents and adults. Eat Behav. 2021;42:101538. 34247036 10.1016/j.eatbeh.2021.101538 PMC8518978

[12] Tang X, Forbush KT, Lui PP. Development and validation of the Chinese-language version of the eating pathology symptoms inventory. Int J Eat Disord. 2015;48(7):1016–23. 26171958 10.1002/eat.22423

[13] Sahlan RN, Blomquist KK, Bodell LP. Psychometric properties of the Farsi version of the eating pathology symptoms inventory (F-EPSI) among Iranian university men and women. J Eat Disorders. 2022;10:67. 10.1186/s40337-022-00587-w PMC9082464 35534863

[14] Sahlan RN, Saunders JF, Fitzsimmons-Craft EE. Validation of a Farsi version of the eating pathology symptoms inventory (F-EPSI) among Iranian adolescents. Eat Weight Disorders. 2023;28:33. 10.1007/s40519-023-01561-4 PMC10042940 36971859

[15] Birgegård A, Isomaa R, Monell E, Bjureberg J. Validation of the eating pathology symptoms inventory (EPSI) in Swedish adolescents. J Eat Disorders. 2024;12:68. 10.1186/s40337-024-01027-7 PMC11129359 38802891

[16] Forbush KT, Bohrer BK, Hagan KE, Chapa DAN, Perko V, Richson B, et al. Development and initial validation of the eating pathology symptoms Inventory–Clinician-Rated version (EPSI-CRV). Psychol Assess. 2020;32(10):943–55. 32718162 10.1037/pas0000820

[17] Koltun KJ, Bird MB, Lovalekar M, Martin BJ, Mi Q, Nindl BC. Changes in eating pathology symptoms during initial military training in men and women and associations with BMI and injury risk. Eat Behav. 2023;48:101687. 36463664 10.1016/j.eatbeh.2022.101687

[18] Hilbert A, Tuschen-Caffier B. Eating disorder Examination–Questionnaire. Tübingen: dgvt; 2016.

[19] Meule A. Diagnostik von Essverhalten. Göttingen: Hogrefe; 2020.

[20] Nosek BA, Errington TM. What is replication? PLoS Biol. 2020;18(3):e3000691. 32218571 10.1371/journal.pbio.3000691 PMC7100931

[21] Fairburn CG, Beglin SJ. Assessment of eating disorders: interview or self-report questionnaire? Int J Eat Disord. 1994;16:363–70. 7866415

[22] Evans C, Dolan B. Body shape questionnaire: derivation of shortened alternate forms. Int J Eat Disord. 1993;13(3):315–21. 8477304 10.1002/1098-108x(199304)13:3<315::aid-eat2260130310>3.0.co;2-3

[23] Pook M, Tuschen-Caffier B, Brähler E. Evaluation and comparison of different versions of the body shape questionnaire. Psychiatry Res. 2008;158(1):67–73. 18037499 10.1016/j.psychres.2006.08.002

[24] Lovibond PF, Lovibond SH. The structure of negative emotional States: comparison of the depression anxiety stress scales (DASS) with the Beck depression and anxiety inventories. Behav Res Ther. 1995;33(3):335–43. 7726811 10.1016/0005-7967(94)00075-u

[25] Nilges P, Essau C. Die Depressions-Angst-Stress-Skalen. Der Schmerz. 2015;29(6):649–57. 26205682 10.1007/s00482-015-0019-z

[26] DiStefano C, Morgan GB. A comparison of diagonal weighted least squares robust Estimation techniques for ordinal data. Struct Equ Model. 2014;21(3):425–38.

[27] Li C-H. Confirmatory factor analysis with ordinal data: comparing robust maximum likelihood and diagonally weighted least squares. Behav Res Methods. 2016;48:936–49. 26174714 10.3758/s13428-015-0619-7

[28] Schermelleh-Engel K, Moosburger H, Müller H. Evaluating the fit of structural equation models: tests of significance and descriptive goodness-of-fit measures. Methods Psychol Res Online. 2003;8:23–74.

[29] McDonald RP. Test theory: A unified treatment. New York, NY: Psychology; 1999.

[30] McNeish D. Thanks coefficient alpha, we’ll take it from here. Psychol Methods. 2018;23(3):412–33. 28557467 10.1037/met0000144

[31] Wilcox RR. The percentage Bend correlation coefficient. Psychometrika. 1994;59(4):601–16.

[32] Mair P, Wilcox R. Robust statistical methods in R using the WRS2 package. Behav Res Methods. 2020;52(2):464–88. 31152384 10.3758/s13428-019-01246-w

[33] Glass GV. Note on rank biserial correlation. Educ Psychol Meas. 1966;26(3):623–31.

[34] Mann HB, Whitney DR. On a test of whether one of two random variables is stochastically larger than the other. Annals Math Stat. 1947;18(1):50–60.

[35] Benjamin DJ, Berger JO, Johannesson M, Nosek BA, Wagenmakers EJ, Berk R, et al. Redefine statistical significance. Nat Hum Behav. 2018;2:6–10. 30980045 10.1038/s41562-017-0189-z

[36] Kliem S, Mößle T, Zenger M, Strauß B, Brähler E, Hilbert A. The eating disorder examination-questionnaire 8: A brief measure of eating disorder psychopathology (EDE-Q8). Int J Eat Disord. 2016;49:613–6. 26711183 10.1002/eat.22487

[37] Davis C, Brewer H, Ratusny D. Behavioral frequency and psychological commitment: necessary concepts in the study of excessive exercising. J Behav Med. 1993;16:611–28. 8126715 10.1007/BF00844722

[38] Zeeck A, Schlegel S, Giel KE, Junne F, Kopp C, Joos A, et al. Validation of the German version of the commitment to exercise scale. Psychopathology. 2017;50:146–56. 28241132 10.1159/000455929

[39] Thome JL, Espelage DL. Obligatory exercise and eating pathology in college females: replication and development of a structural model. Eat Behav. 2007;8:334–49. 17606231 10.1016/j.eatbeh.2006.11.009

[40] Dittmer N, Voderholzer U, Mönch C, Cuntz U, Jacobi C, Schlegl S. Efficacy of a specialized group intervention for compulsive exercise in inpatients with anorexia nervosa: a randomized controlled trial. Psychother Psychosom. 2020;89:161–73. 32036375 10.1159/000504583

[41] Dittmer N, Voderholzer U, von der Mühlen M, Marwitz M, Fumi M, Mönch C, et al. Specialized group intervention for compulsive exercise in inpatients with eating disorders: feasibility and preliminary outcomes. J Eat Disorders. 2018;6(27):1–11. 10.1186/s40337-018-0200-8 PMC6131908 30214803

[42] Schlegl S, Vierl L, Kolar DR, Dittmer N, Voderholzer U. Psychometric properties of the compulsive exercise test in a large sample of female adolescent and adult inpatients with anorexia nervosa and bulimia nervosa. Int J Eat Disord. 2022;55(4):494–504. 35199345 10.1002/eat.23694

[43] Meule A, Schrambke D, Furst Loredo A, Schlegl S, Naab S, Voderholzer U. Inpatient treatment of anorexia nervosa in adolescents: a one-year follow up study. Eur Eat Disorders Rev. 2021;29:165–77. 10.1002/erv.2808 33230832

[44] Fabry FM, Schlegl S, Voderholzer U, Kolar DR, Meule A. Psychometric properties of the Commitment to Exercise Scale in inpatients with anorexia nervosa and bulimia nervosa. Poster presented at the 4th German Psychotherapy Congress, Berlin, Germany. 2025. 10.1002/erv.70099 41817418

[45] Kroenke K, Spitzer RL, Williams JBW, Löwe B. An ultra-brief screening scale for anxiety and depression: the PHQ–4. Psychosomatics. 2009;50(6):613–21. 19996233 10.1176/appi.psy.50.6.613

[46] Löwe B, Wahl I, Rose M, Spitzer C, Glaesmer H, Wingenfeld K, et al. A 4-item measure of depression and anxiety: validation and standardization of the patient health Questionnaire-4 (PHQ-4) in the general population. J Affect Disord. 2010;122(1):86–95. 19616305 10.1016/j.jad.2009.06.019

[47] Freeston MH, Rhéaume J, Letarte H, Dugas MJ, Ladouceur R. Why do people worry? Pers Indiv Differ. 1994;17(6):791–802.

[48] Gerlach AL, Andor T, Patzelt J. Die bedeutung von unsicherheitsintoleranz für die generalisierte angststörung modellüberlegungen und entwicklung einer Deutschen version der unsicherheitsintoleranz-Skala. Z Für Klinische Psychologie Und Psychother. 2008;37(3):190–9.

[49] Patarinski AGG, Adams LM, Fischer S. Examining the relationship of eating disorder symptoms and perfectionism in men and women using two assessments of eating pathology. Eat Behav. 2023;48:101704. 36724674 10.1016/j.eatbeh.2023.101704

[50] Brown M, Robinson L, Campione GC, Wuensch K, Hildebrandt T, Micali N. Intolerance of uncertainty in eating disorders: a systematic review and meta-analysis. Eur Eat Disorders Rev. 2017;25(5):329–43. 10.1002/erv.2523 28544668

[51] Kesby A, Maguire S, Brownlow R, Grisham JR. Intolerance of uncertainty in eating disorders: an update on the field. Clin Psychol Rev. 2017;56:94–105. 28710918 10.1016/j.cpr.2017.07.002

[52] Abber SR, Becker KR, Stern CM, Palmer LP, Joiner TE, Breithaupt L, et al. Latent profile analysis reveals overlapping ARFID and shape/weight motivations for restriction in eating disorders. Psychol Med. 2024;54(11):2956–66. 38801097 10.1017/S003329172400103X PMC11599471

[53] Haberman SJ. When can subscores have value? J Educational Behav Stat. 2008;33(2):204–29.

[54] Dai S, Svetina D, Wang X. Reporting subscores using R: a software review. J Educational Behav Stat. 2017;42(5):617–38.

[55] Cohen J. Statistical power analysis for the behavioral sciences. 2nd ed. Lawrence Erlbaum Associates; 1988.

